# Novel multi-component crystals of berberine with improved pharmaceutical properties

**DOI:** 10.1107/S2052252522010983

**Published:** 2023-01-01

**Authors:** Guoshun Zhang, Xirui Yang, Xiaoqing Shang, Wei Han, Fengfeng Wang, Shurong Ban, Shuqiu Zhang

**Affiliations:** a Department of Pharmacy, Shanxi Medical University, Taiyuan 030001, People’s Republic of China; b Department of Pharmacy, Shanxi Health Vocational College, Taiyuan 030001, People’s Republic of China; c National Institutes for Food and Drug Control, Beijing 100050, People’s Republic of China; University of Iowa, USA

**Keywords:** solubility, crystal stability, multi-stoichiometric solids, berberine

## Abstract

As an extremely popular natural product, berberine (BER) is mainly used for gastroenteritis and diarrhoea caused by bacteria. Research has also revealed the potent and extensive pharmacological properties of BER including its anti-arrhythmic, anti-tumour, anti-inflammatory and hypoglycemic activities and so on; therefore, BER is a promising drug for further development. However, its commercial form with hydro­chloride exhibits poor stability and solubility, which are detrimental to its clinical therapeutic effects. For these purposes, the salt form was regulated via the reactive crystallization of 8-hy­droxy-7,8-di­hydro­berberine (8H-HBER) with five pharmaceutically suitable organic acids including malonic acid (MA), l-tartaric acid (lTA), d-tartaric acid (dTA), dl-tartaric acid (dlTA) and citric acid (CA), resulting in the six novel solid forms 1BER-1lTA-1W, 1BER-1dTA-1W, 1BER-1dlTA and 2BER-2CA as well as two rare multi-stoichiometric solid forms 1BER-1MA and 1BER-2MA-2W. The preparation of the multi-stoichiometric products was greatly influenced by both the crystallization solvent type and the molar ratio of reactants. The structures of these multi-component solid forms were determined using single-crystal X-ray diffraction and further characterized by powder X-ray diffraction, thermal analysis and Fourier transform infrared spectroscopy. Stability experiments showed that all samples prepared had superior physical stability under high temperature and high humidity. Furthermore, dissolution experiments demonstrated that the maximum apparent solubilities (MAS) of all the products were significantly improved compared with the commercial form of BER in dilute hydro­chloric solution (pH = 1.2). In particular, the MAS of 1BER-1MA in dilute hydro­chloric solution is as high as 34 times that of the commercial form. In addition, it is preliminarily confirmed that the MAS of the samples prepared in pure water and dilute hydro­chloric solution is primarily influenced by a combination of factors including the packing index, intermolecular interactions, affinity of the counter-ion to the solvent, the molar ratio of the drug to counter-ion in the product and the common ion effect. These novel solids are potential candidates for BER solid forms with improved oral dosage design and may prompt further development.

## Abstract 2

Simultaneous improvements in stability and solubility of six novel multi-component solid forms synthesized via the reactive crystallization of 8-hydroxy-7,8-dihydroberberine with a number of pharmaceutical acids were observed in comparison with the commercial form of berberine. The factors affecting solubility were determined to be cumulative contributions of the affinity of the counter-ion to the solvent, the packing index, intermolecular interactions, the molar ratio of drug to counter-ion in the product and the common ion effect.

## 1. Introduction

Berberine (BER) extracted from *Coptidis rhizoma* (Huang *et al.*, 2020 ▸) is an iso­quinoline alkaloid used in the clinical treatment of gastroenteritis and diarrhoea caused by bacteria (Shao *et al.*, 2020 ▸; Vishnoi *et al.*, 2021 ▸). Recently, BER has gained increasing attention and is the focus of hundreds of papers every year owing to its extensive pharmacological properties such as anti-arrhythmic failure (Chen *et al.*, 2014 ▸), anti-platelet aggregation (Zhang *et al.*, 2016 ▸), anti-cerebral ischemia (Chai *et al.*, 2013 ▸), anti-tumour (Wang *et al.*, 2020 ▸), anti-viral (Warowicka *et al.*, 2020 ▸), anti-inflammatory (Wu *et al.*, 2018 ▸) and hypoglycemic activities (Yin *et al.*, 2017 ▸) *etc.* Hence great efforts have been devoted by researchers to exploit these properties, with new directions for clinical application.

**Figure 1:**
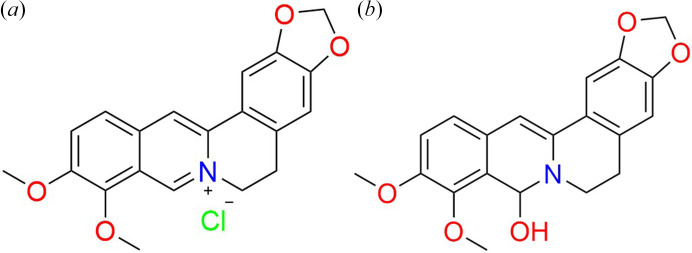
(*a*) Berberine hydro­chloride. (*b*) 8-hy­droxy-7,8-di­hydro­berberine.

The most common commercially available salt form of BER is its hydro­chloride [Fig. 1 ▸(*a*)]. It is reported that BER hydro­chloride exists in four solid forms: anhydrate, monohydrate, dihydrate (1BER-1HCl-2W) and tetrahydrate (1BER-1HCl-4W) (Nakagawa *et al.*, 1978 ▸; Yoshimatsu *et al.*, 1981 ▸). The anhydrate and monohydrate are remarkably hygroscopic and readily convert to the dihydrate at 12% humidity. Humidity >70% would further induce a phase transformation from the dihydrate to the tetrahydrate. Hence, the commercial form of BER hydro­chloride is usually a mixture (1BER-1HCl-M) of its dihydrate and tetrahydrate (Tong *et al.*, 2009 ▸). Variation in the environment may also promote the solid-state transformation of commercially available BER. However, changes in the solid form would not be beneficial for its clinical effects. Moreover, bioavailability of 1BER-1HCl-M is extremely low (<1%), partly due to its poor solubility (Liu, *et al.*, 2016 ▸; Sahibzada, *et al.*, 2020 ▸), which hinders further development.

To overcome the aforementioned drawbacks, one of the most effective strategies for ionizable BER is the modification of its salt form. A Cambridge Structural Database (CSD) search combined with a literature retrieval showed that there are more than 30 hits for BER, and these solid forms were mainly obtained from its hydro­chloride used as the reactant (Allen, 2002 ▸; Deng *et al.*, 2018 ▸; Lu *et al.*, 2018 ▸; Wang *et al.*, 2016 ▸, 2020 ▸, 2021 ▸; Yang *et al.*, 2020 ▸). As a result, the stability issue was indeed resolved, but no substantial improvement in solubility of the products was achieved compared with 1BER-1HCl-M; the solubilities of some of the products such as BER acesulfame and BER saccharine are lower than that of 1BER-1HCl-M (Wang *et al.*, 2016 ▸).

The solubility of a drug is related to a wide variety of factors, leading to a limited understanding of the relationship between its structure and solubility. Recently, many attempts have been made to elucidate this relationship. To the best of our knowledge, the main influencing factors include the packing index (Zhang *et al.*, 2020 ▸), intermolecular interactions (Zhao *et al.*, 2020 ▸), lattice energy (Guo *et al.*, 2018 ▸), the affinity of the counter-ion to the solvent (Ren *et al.*, 2021 ▸), the pH of the solution (Alhalaweh, *et al.*, 2012 ▸), the molar ratio of the drug to counter-ion in the product (Yu *et al.*, 2021 ▸), the common ion effect (Devarapalli *et al.*, 2021 ▸) and so on. A combination of these factors has frequently been found to affect the solubility of a drug.

To acquire a solid form of BER with the desired properties, we anticipated that the reactants would need to be altered. In view of the reversible transformation between the free BER quaternary amine base and 8-hy­droxy-7,8-di­hydro­berberine [8H-HBER, Fig. 1 ▸(*b*)], 8H-HBER serving as the stable solid form (Dostál *et al.*, 2004 ▸), we selected to react 8H-HBER with a number of pharmaceutical acids for the first time. MA, possessing the optimum solubility of the eight common carb­oxy­lic acids reported, was also employed in the study by Apelblat & Manzurola (1987 ▸). Additionally, the effect of chirality of the counter-ion on solubility was investigated with TA, the model acids included l-TA, d-TA and racemic dlTA. Moreover, CA with a solubility that is almost equivalent to lTA was also employed to form a salt with BER in order to evaluate the impact of the counter-ion on the solubility of the resultant products.

The reactive crystallizations of 8H-HBER with the selected acids yielded six novel solid forms of BER including 1BER-1MA, 1BER-2MA-2W, 1BER-1lTA-1W, 1BER-1dTA-1W, 1BER-1dlTA and 2BER-2CA. All the multi-component solid forms with the exception of 1BER-1dlTA were confirmed by single-crystal X-ray diffraction (SCXRD), and further comprehensively characterized using powder X-ray diffraction (PXRD), thermal analysis and Fourier transform infrared spectroscopy (FT-IR). Moreover, stability and solubility of the samples obtained were evaluated and compared with 1BER-1HCl-M. Furthermore, the relationships between the structures of the products and their solubilities were tentatively clarified.

## 2. Methods

### 2.1. Materials

Commercial BER hydro­chloride and BER hydrogen sulfate were purchased from Baoji Fangsheng Biological Development Co. Ltd (Shanxi, China). 8H-HBER (dark yellow powder) was prepared in our laboratory, and its purification confirmed by HPLC was >98%. All other chemicals were of analytical grade and were provided by Sinopharm Chemical Reagent Company Ltd (Shanghai, China). Redistilled water was used throughout the study.

### 2.2. Crystallization

#### 2.2.1. Preparation of 8H-HBER

After complete dissolution of 200 mg of BER hydrogensulfate in water (45 ml), a saturated aqueous solution of NaOH was added until no further precipitation occured. Then, the mixture was extracted three times with 20 ml di­ethyl ether. The di­ethyl ether was removed under reduced pressure to obtain the residual solid sample. Finally, the solid sample collected was dissolved in methanol at 323 K and left at ambient temperature to evaporate. After five days, 8H-HBER precipitated and was analyzed using FT-IR; the FT-IR spectrum was consistent with a previous report (Dostál *et al.*, 2004 ▸).

#### 2.2.2. Preparation of 1BER-1MA and 1BER-2MA-2W

After complete dissolution of 50 mg of the 8H-HBER and MA mixture (molar ratio 1:1 or 1:2) in the solvents listed in Table S1 of the supporting information at 323 K, the solution was filtered and cooled to room temperature. After five days, needle-like crystals were harvested.

#### 2.2.3. Preparation of 1BER-1lTA-1W and 1BER-1dTA-1W

After complete dissolution of 50 mg of the 8H-HBER and acid mixture (molar ratio 1:1) in the mixed acetone–water solvent (*v*/*v* = 3 ml:4 ml) at 323 K, the solution was filtered and cooled to room temperature. After four days, needle-like crystals were harvested.

#### 2.2.4. Preparation of 1BER-1dlTA

After complete dissolution of 50 mg of the 8H-HBER and dlTA mixture (molar ratio 1:1) in the acetone–water solvent (*v*/*v* = 5 ml:5 ml) at 323 K, the solution was filtered and cooled to room temperature. After four days, the product was prepared.

#### 2.2.5. Preparation of 2BER-2CA

After complete dissolution of 50 mg of the 8H-HBER and CA mixture (molar ratio 1:1) in the acetone–water solvent (*v*/*v* = 1.5 ml:2.5 ml) at 323 K, the solution was filtered and cooled to room temperature. After two days, needle-like crystals were harvested.

### 2.3. Single crystal X-ray diffraction

SCXRD data for the harvested crystals were collected on a Bruker APEX-II D8 Venture area diffractometer (Bruker, Germany). The crystal structures were solved via direct methods and refined by a full-matrix least-squares procedure against *F* ^2^ using *OLEX2* (Dolomanov *et al.*, 2009 ▸) and *SHELXTL* (Sheldrick, 2008 ▸) with anisotropic displacement parameters (ADPs) for non-hydrogen atoms. Hydrogen atoms bonded to nitrogen or oxygen were determined by the experimental electron density map, and their isotropic displacement parameters and positions were refined freely. All other hydrogen atoms were located in geometrically calculated positions and refined using a riding model.

Hirshfeld surfaces formed by the equal electron density point between the investigated molecule and its surrounding molecules were obtained by *Crystal Explorer* (version 3.1; McKinnon *et al.*, 2004 ▸) based on the SCXRD data. After, various intermolecular interactions among the different crystal structures were quantitatively explored and compared.

### 2.4. Powder X-ray diffraction

PXRD experiments were performed on a Rigaku D/max-2550 diffractometer with Cu *K*α radiation (Rigaku, Tokyo, Japan) at 40 kV and 150 mA at 293 K. PXRD patterns were recorded at a constant 8° min^−1^ scan rate in the 2θ range 5–60°.

### 2.5. Thermal analysis

Differential scanning calorimetry (DSC) and thermogravimetric analysis (TGA) were conducted on a ZCT-B DSC/TGA calorimeter (Jingyi Gaoke Instrument Co. Ltd, Beijing, China). The accurately weighed samples in open aluminium oxide cells were heated from 313 to 573 K at a constant rate of 10 K min^−1^.

### 2.6. Fourier transform infrared spectroscopy analysis

FT-IR analysis was performed using a Nicolet Nexus IS5 FT-IR with an attenuated total reflectance (ATR) accessory (Thermo-Nicolet, Madison, USA). The scan range was set from 500 to 4000 cm^−1^ with a 4 cm^−1^ resolution.

### 2.7. Stability test

Samples were subjected to accelerated conditions of high temperature (313 K and/or 333 K, 40% ± 5%) and high humidity (298 K, 90% ± 5%) for 10 days according to the *Chinese Pharmacopoeia* (Pharmacopoeia committee of the People’s Republic of China, 2020 ▸) and further analysed via FT-IR to determine whether the samples had changed.

### 2.8. Dissolution experiments

All solid-state samples were sieved through a 100-mesh screen before dissolution experiments were performed. Then, a dissolution experiment was conducted on a constant-temperature oscillator (ZWY-103B, Shanghai Zhicheng Analytical Instrument Manufacturing Co. Ltd, China). Approximately 5 mg of 1BER-1HCl-M (accurately weighed) was dissolved in 100 ml hydro­chloric acid solution (pH = 1.2). Seven primary standard solutions were prepared by suitably diluting the stock solutions to generate a calibration curve. Excess amounts of seven different samples including 1BER-1MA, 1BER-2MA-2W, 1BER-1lTA-1W, 1BER-1dTA-1W, 1BER-1dlTA, 2BER-2CA and 1BER-1HCl-1M were added to 30 ml pure water or dilute hydro­chloric acid solution (pH = 1.2). The solutions were shaken at 310 K for 48 h at a speed of 100 rpm. The suspensions were removed at 5, 10, 20, 30 and 45 min; and 1, 1.5, 2, 3, 24 and 48 h, and filtered through 0.2 µm membrane filters. Finally, the liquids were diluted appropriately and measured spectrophotometrically to determine the UV absorbance (L8, Shanghai Yidian Analytical Instrument Co. Ltd, China). The detection wavelength was set to 340 nm. Moreover, the undissolved samples were collected at 3 and 48 h for FT-IR analysis.

## 3. Results and discussion

### 3.1. Crystallization

The stoichiometric ratio of ionizable drug and counter-ion in the resulting product is commonly controlled by the molar ratio of reactants. As expected, 1BER-1MA is prepared via the reactive crystallization of 8H-HBER with MA in a 1:1 molar ratio in the ethanol and water mixed solvent system. However, the 1:2 molar ratio of 8H-HBER to MA also produces 1BER-1MA when the volume ratio of water and ethanol was below 1:9. 1BER-2MA-2W could be harvested from the mixed solvent system of acetone and water, regardless of the 1:1 or 1:2 molar ratio of 8H-HBER and MA. Crystallizations of 1BER-1MA and 1BER-2MA-2W were influenced by the crystallization solvent type as well as the molar ratio of reactants. Thus, preparation of the desired stoichiometric salt required the appropriate crystallization solvents as well as molar ratio of reactants.

### 3.2. Structural analysis

Five novel crystal structures were determined using SCXRD. The crystallographic parameters and strong hydrogen-bond geometries of the five crystals are listed in Tables 1 ▸ and 2 ▸. Loss of a proton derived from the acid occurred in each crystal. Hence, these samples were termed salts. However, an additional neutral MA molecule was also included in 1BER-2MA-2W, which can be classified as a salt cocrystal.

#### 3.2.1. BER-1MA

**Figure 2:**
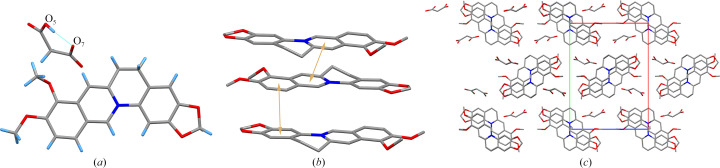
(*a*) Hydrogen bonds between molecules in 1BER-1MA. (*b*) π–π interactions between molecules in 1BER-1MA (hydrogen atoms have been omitted). (*c*) Three-dimensional structure of 1BER-1MA (hydrogen atoms have been omitted).

1BER-1MA crystallized in the monoclinic space group *P*2_1_/*c* with one BER cation and one MA anion in the asymmetric unit. The intramolecular hydrogen bond O_5_—H⋯O_7_ in the MA anion generates a ring-like structure [Fig. 2 ▸(*a*)]. However, no intermolecular hydrogen bonds were observed in 1BER-1MA. π–π interactions in 1BER-1MA were formed between two benzene rings (C_1_—C_2_—C_3_—C_4_—C_5_ and C_10_—C_11_—C_14_—C_15_—C_16_) [shortest centre-to-centre distance: 3.549 (3) Å] and between the six-membered rings (N_1_—C_8_—C_7_—C_6_—C_5_—C_9_) [shortest centre-to-centre distance: 3.795 (3) Å] to construct a layer-like structure [Fig. 2 ▸(*b*)]. The layer-like structures depend on van der Waals forces to create a three-dimensional structure with MA anions scattered in the voids [Fig. 2 ▸(*c*)].

The unit-cell parameters of 1BER-1MA were evidently affected by the measured temperature. The 6.844 (1) Å *a* axis and 19.545 (1) Å *b* axis at 100 K increased to 6.943 (1) Å and 19.818 (2) Å at 293 K, respectively, whereas the *c* axis showed almost no change. This implies that the peak positions of 1BER-1MA between the simulated PXRD pattern based on SCXRD data at 100 K and the experimental PXRD pattern collected at 293 K would be different.

#### 3.2.2. BER-2MA-2W

**Figure 3:**
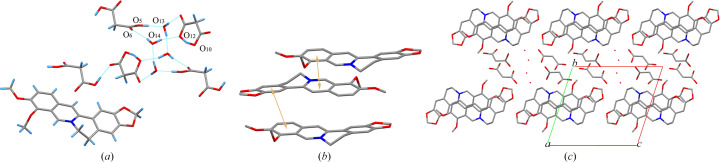
(*a*) Hydrogen bonds between molecules in 1BER-2MA-2W. (*b*) π–π interactions between molecules in 1BER-2MA-2W (hydrogen atoms have been omitted). (*c*) Three-dimensional structure of 1BER-2MA-2W (hydrogen atoms have been omitted).

1BER-2MA-2W crystallized in the triclinic space group *P* 1 with one BER cation, one MA anion, one neutral MA molecule and two water molecules in the asymmetric unit. Similar to the MA anion in 1BER-1MA, the intramolecular hydrogen bond O_8_—H_8_⋯O_9_ in 1BER-2MA-2W was generated to form a ring motif [Fig. 3 ▸(*a*)]. Only one of the two MA molecules in the asymmetric unit was deprotonated. Thus, 1BER-2MA-2W was regarded as a salt cocrystal. Two water molecules, one MA anion and one MA molecule were connected via the hydrogen bonds O_5_—H⋯O_13_ (−*x* + 1, −*y* + 1, −*z*), O_13_—H⋯O_11_ (−*x* + 1, −*y* + 1, −*z* − 1), O_14_—H⋯O_6_ (−*x* + 2, −*y*, −*z* − 1) and O_14_—H⋯O_12_ (−*x* + 2, −*y*, −*z* − 1) to form a 12-membered ring motif. Moreover, two 12-membered ring motifs were bonded to each other via the hydrogen bond O_13_—H⋯O_14_ (−*x* + 2, −*y* + 1, −*z* − 1). Furthermore, the hydrogen-bonded network was extended via the hydrogen bond O_8_—H⋯O_9_ to generate a chain-like structure. π–π interactions in 1BER-2MA-2W [Fig. 3 ▸(*b*)] were formed between two benzene rings (C_1_—C_2_—C_3_—C_4_—C_5_—C_6_ and C_10_—C_11_—C_14_—C_15_—C_16_—C_17_) [shortest centre-to-centre distance: 3.6476 (10) Å] and between the six-membered ring (N_1_—C_8_—C_7_—C_6_—C_5_—C_9_) and the benzene ring (C_1_—C_2_—C_3_—C_4_—C_5_—C_6_) [shortest centre-to-centre distance: 3.8355 (10) Å] to construct a layer-like structure. Ultimately, the chain-like structures and layer-like structures that rely on van der Waals forces were assembled into a three-dimensional structure with hydro­phobic zones and hydro­philic zones in an alternate arrangement [Fig. 3 ▸(*c*)].

#### 3.2.3. BER-1lTA-1W and 1BER-1dTA-1W

**Figure 4:**
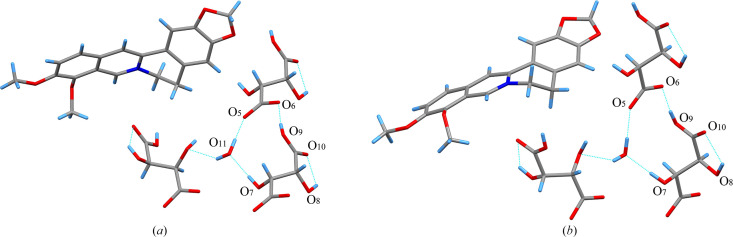
Hydrogen bonds between molecules in (*a*) 1BER-1l-TA-1W and (*b*) 1BER-1dTA-1W.

**Figure 5:**
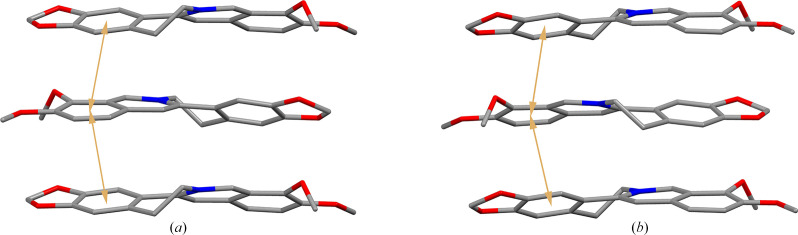
π–π interactions among molecules in (*a*) 1BER-1lTA-1W and (*b*) 1BER-1dTA-1W (hydrogen atoms have been omitted).

**Figure 6:**
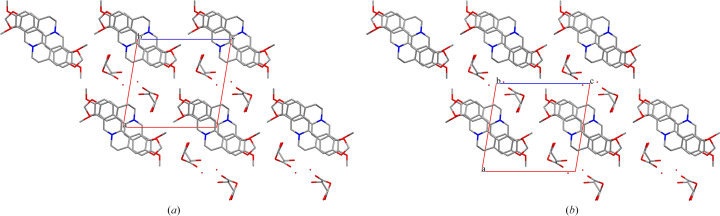
Three-dimensional structures of (*a*) 1BER-1l-TA-1W and (*b*) 1BER-1dTA-1W (hydrogen atoms have been omitted).

TA molecules with distinct chirality were employed in the salt formation process. However, the products obtained with 1BER-1lTA-1W and 1BER-1dTA-1W have similar cell parameters and crystal structure. This indicates that the two samples would have almost the same PXRD pattern, FT-IR spectra and TGA/DSC curves. They crystallized in the triclinic space group *P*2_1_ with one BER cation, one TA anion and one water molecule in the asymmetric unit. Flack parameters for 1BER-1lTA-1W and 1BER-1dTA-1W below 0.3 were 0.05 (6) and 0.11 (4), respectively. This implies that the absolute configuration of the two crystals could be determined. The intramolecular hydrogen bond O_8_—H⋯O_10_ in the TA anion generates a five-membered ring-like motif (Fig. 4 ▸). Each water molecule is bonded to three TA anions around it via three hydrogen bonds, O_7_—H⋯O_11_, O_11_—H⋯O_5_ and O_11_—H⋯O_7_ to form a chain-like structure. The connection is further strengthened via the hydrogen bond O_9_—H⋯O_6_ to form a 12-membered ring-like structure. π–π interactions in 1BER-1lTA-1W and 1BER-1dTA-1W (Fig. 5 ▸) exist between the two benzene rings (C_1_—C_2_—C_3_—C_4_—C_5_—C_6_ and C_10_—C_11_—C_14_—C_15_—C_16_—C_17_) [shortest centre-to-centre distances: 3.5772 (12) Å and 3.5744 (12)] to construct a layer-like structure. The layer-like structures assemble via van der Waals forces into a three-dimensional structure with hydro­philic chain-like structures scattered in the voids (Fig. 6 ▸).

#### 3.2.4. BER-2CA

**Figure 7:**
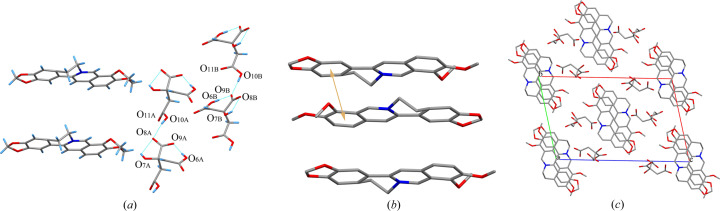
(*a*) Hydrogen bonds between molecules in 2BER-2CA. (*b*) π–π interactions between molecules in 2BER-2CA (hydrogen atoms have been omitted). (*c*) Three-dimensional structure of 2BER-2CA (hydrogen atoms have been omitted).

2BER-2CA crystallized in the triclinic space group *P* 1 with two BER cations and two CA anions in the asymmetric unit. Both BER cations and CA anions could be divided into two types in 2BER-2CA. The C_12B_—C_13B_ group in one type of BER cations is disordered over two positions with 0.53:0.47. The disordered O_11A_ is split over two positions with 0.54:0.46, also observed in one type of the CA anions. Similar intramolecular hydrogen bonds O_6_—H⋯O_9_ and O_7_—H⋯O_8_ form in both types of CA anions to construct five- and seven-membered ring-like motifs [Fig. 7 ▸(*a*)]. The intermolecular hydrogen bond was shaped to yield a chain-like structure among the same type of CA anions, but with O_10A_—H⋯O_8A_ for one type CA anions and O_10B_—H… O_9B_ for the other. π–π interactions in 2BER-2CA were generated between two benzene rings (C_1_—C_2_—C_3_—C_4_—C_5_—C_6_ and C_10_—C_11_—C_14_—C_15_—C_16_—C_17_) [shortest centre-to-centre distance: 3.797 (3) Å] resulting in a layer-like structure [Fig. 7 ▸(*b*)]. Similar to 1BER-1LTA-1W and 1BER-1DTA-1W, a three-dimensional structure with hydro­philic chain-like structures scattered in the voids assembled by the layer-like structures were formed [Fig. 7 ▸(*c*)].

#### 3.2.5. Packing coefficient

The packing coefficient was calculated using the *PLATON* software (Spek, 2009 ▸) based on SCXRD data according to the following equation (Price *et al.*, 2006 ▸): *C*_k_ = *ZV*_mol_ *V*_cell_^−1^, where *Z*, *V*_mol_ and *V*_cell_ represent the number of molecules in the unit cell, the molecular volume (Å^3^) and the volume of the unit cell (Å^3^), respectively.

The percentage of void space in molecular crystals was negatively related to the packing coefficient. A large packing coefficient for the crystal indicates a tight packing among molecules, suggesting lower solubility. The packing coefficients for 1BER-1MA, 1BER-2MA-2W, 1BER-1lTA-1W, 1BER-1dTA-1W and 2BER-2CA were determined to be 73.8, 73.0, 75.3, 75.6 and 69.6%, respectively.

### 3.3. Hirshfeld surface analysis

**Figure 8:**
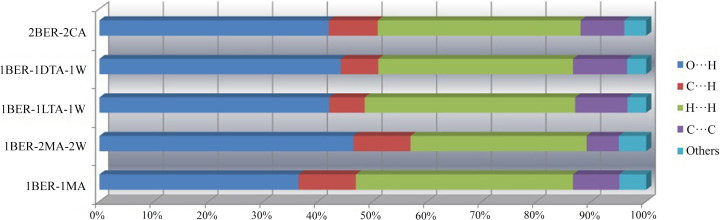
Summary of intermolecular interactions obtained from the Hirshfeld surface area for the five multi-component crystals.

The various intermolecular interactions quantitatively obtained from the Hirshfeld surface analysis could reveal the strength of the connection among molecules to some extent, which may be reflected in the solubility and used for rationalizing the solid-state formation of a drug (Sanphui *et al.*, 2015 ▸). Intermolecular interactions of the samples obtained are summarized in Fig. 8 ▸. It is clear that the O⋯H, C⋯H, H⋯H and C⋯C interactions contribute significantly to the Hirshfeld surfaces. An increasing proportion of the strong O⋯H interactions – with 46.4% in 1BER-2MA-W – was observed because two water molecules and one additional MA molecule are incorporated into the crystal structure compared with 36.4% in 1BER-1MA, and there is no obvious difference in packing coefficient between 1BER-1MA and 1BER-2MA-2W. This implies a stronger connection between the molecules in 1BER-2MA-2W. Thus, it is favourable for the formation of 1BER-2MA-2W over 1BER-1MA when the water content in solution and molar ratio of MA to BER increase to a certain extent.

### 3.4. PXRD analysis

Significant differences between the solid forms obtained could be effectively detected by PXRD, with the exception of 1BER-1lTA-1W and 1BER-1dTA-1W (Figs. S1–S6). The experimental powder patterns of the crystals were well matched with their theoretical powder patterns calculated using *Mercury* [version 3.0; Cambridge Crystallographic Data Centre, Cambridge, UK (Macrae *et al.*, 2006 ▸)], implying high purity of samples. The main experimental peak positions observed were 7.500, 8.873, 10.752, 12.122, 17.278, 17.832, 18.773, 18.997, 19.960, 25.688, 26.895 and 27.548° for 1BER-1MA; 6.721, 7.249, 14.459, 17.583, 19.628, 21.758, 25.298 and 26.402° for 1BER-2MA-2W; 6.971, 7.352, 9.236, 13.986, 14.724, 15.194, 24.927 and 26.629° for 1BER-1dTA-1W; 7.028, 7.399, 9.297, 14.043, 14.784, 15.252, 24.952 and 26.670° for 1BER-1lTA-1W; 7.007, 8.356, 13.719, 15.109, 16.705, 23.294 and 25.193° for 1BER-1dl-TA; and 6.701, 8.074, 13.332, 14.761, 16.297, 16.829, 24.193 and 27.589° for 2BER-2CA.

The experimental peak positions of 1BER-2MA-2W, 1BER-1lTA-1W, 1BER-1dTA-1W and 2BER-2CA were essentially consistent with the calculated peak positions derived from the SCXRD data collected at 100 K. This suggests that there are no obvious differences in the unit-cell parameters of these crystals between 100 and 293 K. However, the *a* and *b* axes of 1BER-1MA at 100 K relative to 293 K were notably shortened, which caused some peak positions at 100 K to differ from those at 293 K.

### 3.5. Thermal analysis

DSC and TGA curves of the samples obtained are depicted in Figs. S7–S8. The proportions of water weight loss for 1BER-2MA-2W, 1BER-1lTA-1W and 1BER-1dTA-1W were 6.20, 3.55 and 3.65%, respectively. The calculated loss numbers of water for the asymmetric units based on the weight loss values were in accordance with the SCXRD data (Table 3 ▸).

Only one endothermic peak corresponding to melting and decomposition in the DSC plots of 1BER-1MA, 1BER-1dl-TA and 2BER-2CA was observed. However, another endothermic peak was observed in both 1BER-1lTA-1W and 1BER-1dTA-1W that could be attributed to the crystalline water loss. Comparatively, there were four endothermic peaks for 1BER-2MA-2W, which were successively attributed to water molecule loss, uncharged malonic acid loss, solid-state transformation, and melting and decomposition, on the basis of analysing the TGA and PXRD data.

Although every water molecule in 1BER-2MA-2W (similar to that in BER-1lTA-1W and 1BER-1dTA-1W) was also bonded to molecules around it via three hydrogen bonds, the dehydration temperature of 1BER-2MA-2W was evidently lower than BER-1lTA-1W and 1BER-1dTA-1W. The reason for this phenomenon may be the lower melting point of the molecules including the water molecule and malonic acid in 1BER-2MA-2W (which form hydrogen bonds with water molecules), relative to those of the molecules of tartaric acid in BER-1lTA-1W and 1BER-1dTA-1W (which also form hydrogen bonds with water molecules).

After 10 min dehydration at 378 K for 1BER-1lTA-1W and 1BER-1dTA-1W and at 353 K for 1BER-2MA-2W, the samples were characterized by PXRD (Figs. S9–S11), and the results showed that only the sample derived from 1BER-2MA-2W had undergone obvious changes in peak positions before and after dehydration. This signified that the crystal of 1BER-2MA-2W had collapsed following the loss of water to form a new solid (1BER-2MA-2W-AH) with the main experimental peak positions 6.109, 6.397, 7.581, 7.888, 10.447, 12.368, 15.418, 15.950, 16.872, 17.177, 19.243, 22.660, 23.949, 24.273, 25.380, 26.179 and 26.424°, while both 1BER-1lTA-1W and 1BER-1dTA-1W only formed anhydrates without crystal collapse.

The second DSC peak of 1BER-2MA-2W corresponded to a 17.80% weight loss in the TGA curve, almost equivalent to the calculated uncharged malonic acid loss value of 17.94% based on the SCXRD data. A third endothermic peak appeared along with the weight loss of the uncharged malonic acid. We inferred that the weight loss is accompanied by transformation of the solid form. Thus, the 1BER-2MA-2W sample after heating for 10 min at 423 K was characterized by PXRD, and the results suggested that the residual solid was 1BER-1MA.

Melting and decomposition appear to induce the formation of the novel compounds, and the TGA curves of all investigated samples after melting and decomposition seem flat to support this, which suggests that the products may be pure solid phases. Hence, all the products after melting and decomposition were monitored using FT-IR and were found to correspond to berberrubine derived from 1BER-1HCl-M (Iwasa *et al.*, 1996 ▸) when heated for 10 min at 483 K. Therefore, these novel salts could also be employed to prepare berberrubine.

No de-solvent peak was observed for 1BER-1dlTA. Moreover, the weight loss value attributed to melting and decomposition for 1BER-1dlTA was roughly equivalent to that for 1BER-1lTA-1W and 1BER-1dTA-1W. Thus, the components included in 1BER-1dlTA were determined to be BER cations and dlTA anions with the molar ratio 1:1.

### 3.6. FT-IR spectroscopic analysis

The peak position, number and shape in the regions 3200–3800 cm^−1^ and 1700–1800 cm^−1^ attributed to the characteristic OH and C=O absorptions were remarkably different among the samples obtained due to the distinct hydrogen interactions (Fig. S12), with the exception of 1BER-1lTA-1W and 1BER-1dTA-1W. FT-IR could effectively monitor the change in solid forms so was employed to analyse the recrystallization product, the stability of the obtained solid forms and the solid forms of the undissolved samples in the dissolution experiments.

### 3.7. Stability

In this study, the stability of the synthesized products against high humidity and high temperature over 10 days were evaluated. As presented in Table 4 ▸ and Figs. S13–S18, all the samples prepared retained their original solid form except for 1BER-2MA-2W. The sample derived from 1BER-2MA-2W at high temperature was characterized by PXRD and found to be the new solid form 1BER-2MA-2W-AH. Thus, we further investigated the stability of 1BER-2MA-2W at high temperature (313 K) and the results showed that 1BER-2MA-2W remained stable. In comparison with the commercial form of BER, significant enhancement in stability was achieved for the novel solid forms.

### 3.8. Powder dissolution experiments

**Figure 9:**
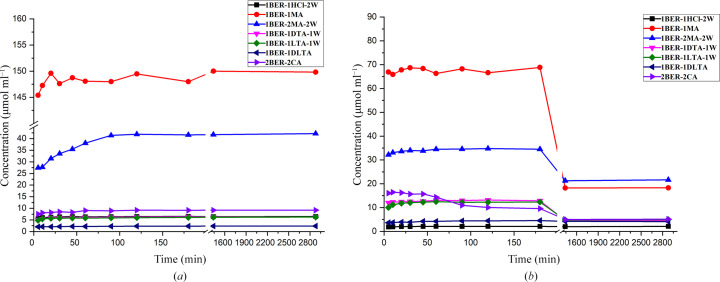
Dissolution profiles of the solid forms obtained in (*a*) pure water and (*b*) dilute hydro­chloric acid solution.

It is widely known that elucidation of the relationship between the solid form of a drug and its solubility is particularly difficult, but it is of great importance for screening an adequate solid form for prescription design. As stated in the introduction, the solubility of a salt is usually controlled by a wide variety of factors such as the packing index, intermolecular interactions, lattice energy, the affinity of the counter-ion to the solvent, the pH of the solution, the common ion effect and so on. Dissolution profiles of the newly obtained samples compared with 1BER-1HCl-M in pure water and dilute hydro­chloric solution (pH = 1.2) are depicted in Fig. 9 ▸.

As presented in Fig. 9 ▸(*a*), the order of maximum apparent solubility (MAS) observed in pure water for the investigated samples was 1BER-1MA with 149.5 µmol ml^−1^ > 1BER-2MA-2W with 41.9 µmol ml^−1^ > 2BER-2CA with 9.2 µmol ml^−1^ > 1BER-1HCl-M with 6.5 µmol ml^−1^ ≃ 1BER-1dTA-1W with 6.2 µmol ml^−1^ ≃ 1BER-1lTA-1W with 6.1 µmol ml^−1^ > 1BER-1dl-TA with 2.3 µmol ml^−1^. Solubilities (the mole fraction solubility) of MA, CA, dTA/lTA and dlTA in water are reported to be 0.2445, 0.1617, 0.1583 and 0.0365, respectively (Apelblat & Manzurola, 1987 ▸; Tan *et al.*, 2016 ▸). It is obvious that the MAS of the novel salts was markedly affected by the affinity of the counter-ion to water. Among these organic acids, MA exhibits the best solubility. Therefore, the MAS of BER malonate was significantly higher than that of the other BER salts. In addition, 1BER-1MA and 1BER-2MA-2W included the same type of counter-ion as malonic acid and had similar packing indexes, but the former displayed better solubility, which may be attributed to the weaker intermolecular interactions with a lower proportion of O⋯H interactions (36.4%) compared with 1BER-2MA-2W (46.4%). In particular, the effect of chirality of TA on the MAS of the final products was clearly related to their solubilities. This phenomenon was in accordance with Wallach’s rule (Brock *et al.*, 1991 ▸; Wang *et al.*, 2012 ▸) which states that racemic crystals have a higher density relative to their chiral counterparts. Thus, the racemic crystals of 1BER-1dl-TA displayed a lower solubility compared with their chiral counterparts 1BER-1dTA-1W and 1BER-1lTA-1W. Solubility of CA was slightly greater than that of dTA/lTA. However, significant improvement in the MAS for 2BER-2CA was achieved compared with 1BER-1lTA-1W and 1BER-1dTA-1W, which resulted from a smaller packing coefficient of 2BER-2CA with 69.6% relative to that of 1BER-1lTA-1W with 75.3% and 1BER-1dTA-1W with 75.6%.

As presented in Fig. 9 ▸(*b*), 1BER-1HCl-M, 1BER-1MA, 1BER-2MA-2W, 1BER-1dTA-1W, 1BER-1lTA-1W, 1BER-1dl-TA and 2BER-2CA achieved MAS in dilute hydro­chloric solution at 2.1, 68.8, 34.8, 13.1, 12.5, 4.5 and 16.4 µmol ml^−1^, respectively. The MAS order in dilute hydro­chloric solution for the samples was in agreement with that in pure water, except for 1BER-1HCl-M with the lowest MAS, which may be the result of the common ion effect. The MAS of all the samples obtained was notably higher than that of the commercial form (1BER-1HCl-M), especially 1BER-1MA with a 33-fold increase.

Given that the solid form is largely related to solubility, all the residual solid forms were analysed after 3 and 48 h by FT-IR (Table 5 ▸). As expected, all the residual solid forms derived from pure water (Figs. S19–S20) showed no difference from their original forms, except for the commercial form of BER, which underwent full conversion to 1BER-1HCl-4W. 1BER-2MA-2W did not convert to 1BER-1MA in pure water. Hence, the common ion effect was not the cause of the lower MAS of 1BER-2MA-2W compared with 1BER-1MA.

The solid form transformation occurred in all the residual solid forms that originated from the dilute hydro­chloric solution (Figs. S21 and S22), but the conversion rate of the solid forms is obviously different among the solid forms obtained. 1BER-1MA, 1BER-2MA-2W and 2BER-2CA underwent complete conversion to 1BER-1HCl-4W after 3 h. In comparison, small amounts of 1BER-1dTA-1W and 1BER-1lTA-1W were converted to 1BER-1HCl-4W after 3 h. No conversion of solid form was observed from the residual sample of 1BER-1dl-TA after 3 h. However, all the residual solid forms that originated from dilute hydro­chloric solution were finally converted to 1BER-1HCl-4W after 48 h. This signifies that there may be a positive correlation between the conversion rate and the MAS of the distinct salts. In addition, owing to solid-state transformation, the apparent solubilities at 48 h of 1BER-1MA, 1BER-2MA-2W, 2BER-2CA, 1BER-1dTA-1W, 1BER-1lTA-1W and 1BER-1dl-TA below their MAS in dilute hydro­chloric solution, were 18.3, 21.6, 4.6, 5.0, 4.1 and 5.1 µmol ml^−1^, respectively. Note that all the investigated salts underwent transformation to 1BER-1HCl-4W, but their final apparent solubilities were different and significantly greater than that of 1BER-1HCl-4W derived from 1BER-1HCl-M. It is inferred that the organic acids replaced by hydro­chloric acid in solution may affect the apparent solubility of 1BER-1HCl-4W.

## 4. Conclusions

Six novel BER multi-component solid forms were successfully synthesized via the reactive crystallization of 8H-HBER with a number of pharmaceutical acids for the first time. Five of the multi-component solid forms including 1BER-1MA, 1BER-2MA-2W, 1BER-1dTA-1W, 1BER-1lTA-1W and 1BER-1CA were structurally elucidated by SCXRD. All the samples obtained were thoroughly characterized by PXRD, FT-IR and thermal analysis. It is inferred from TGA/DSC data of three BER tartrates that the components in 1BER-1dl-TA were determined to be BER cations and racemic TA anions with a molar ratio of 1:1. Among the six products, 1BER-2MA-2W was a salt cocrystal, the rest were classified as salts. The unit-cell parameters of 1BER-1MA were sensitive to temperature. Subsequently, the calculated characteristic diffraction peak positions of 1BER-1MA at 100 K clearly differ from those at 293 K.

Simultaneous improvements in stability and MAS of BER were observed in comparison with the commercial form of BER. All the solid forms obtained were found to be stable under high temperature and high humidity. The MAS values of BER-1MA and 1BER-2MA-2W in pure water, which show an increase of 23- and 6-fold, respectively, are obviously superior to that of the commercial form of BER. The MAS in dilute hydro­chloric solution of all the samples was also far greater than that of the commercial form. Thus, these novel solid forms could be valuable candidates for addressing the low bioavailability and poor stability of BER.

The solubility of a drug relies significantly on its solid form. However, the intrinsic relationship between the solid form of a drug and its solubility is usually unclear and there is a lack of in-depth investigation. In the present study, the factors affecting solubility were determined to be cumulative contributions of the affinity of the counter-ion to the solvent, the packing index, intermolecular interactions, the molar ratio of drug to counter-ion in the product and the common ion effect.

## Supplementary Material

Crystal structure: contains datablock(s) 1BER-1MA, 1BER-1MA-1, 1BER-2MA-2W, 1BER-1DTA-1W, 1BER-1LTA-1W, 2BER-2CA. DOI: 10.1107/S2052252522010983/lq5045sup1.cif

Structure factors: contains datablock(s) 1BER-1MA. DOI: 10.1107/S2052252522010983/lq50451BER-1MAsup2.hkl

Structure factors: contains datablock(s) 1BER-1MA-1. DOI: 10.1107/S2052252522010983/lq50451BER-1MA-1sup3.hkl

Structure factors: contains datablock(s) 1BER-2MA-2W. DOI: 10.1107/S2052252522010983/lq50451BER-2MA-2Wsup4.hkl

Structure factors: contains datablock(s) 1BER-1DTA-1W. DOI: 10.1107/S2052252522010983/lq50451BER-1DTA-1Wsup5.hkl

Structure factors: contains datablock(s) 1BER-1LTA-1W. DOI: 10.1107/S2052252522010983/lq50451BER-1LTA-1Wsup6.hkl

Structure factors: contains datablock(s) 2BER-2CA. DOI: 10.1107/S2052252522010983/lq50452BER-2CAsup7.hkl

Rietveld powder data: contains datablock(s) 1BER-1MA-1. DOI: 10.1107/S2052252522010983/lq50451BER-1MA-1sup8.rtv

Rietveld powder data: contains datablock(s) 1BER-2MA-2W. DOI: 10.1107/S2052252522010983/lq50451BER-2MA-2Wsup9.rtv

Rietveld powder data: contains datablock(s) 1BER-1DTA-1W. DOI: 10.1107/S2052252522010983/lq50451BER-1DTA-1Wsup10.rtv

Rietveld powder data: contains datablock(s) 1BER-1LTA-1W. DOI: 10.1107/S2052252522010983/lq50451BER-1LTA-1Wsup11.rtv

Rietveld powder data: contains datablock(s) 2BER-2CA. DOI: 10.1107/S2052252522010983/lq50452BER-2CAsup12.rtv

Rietveld powder data: contains datablock(s) 1BER-1DLTA. DOI: 10.1107/S2052252522010983/lq50451BER-1DLTAsup13.rtv

Crystallization experiments, DSC, TG, XPRD and IR, stability of the solid phase forms. DOI: 10.1107/S2052252522010983/lq5045sup14.pdf

CCDC references: 2132141, 2132142, 2132143, 2132144, 2132145, 2132146

## Figures and Tables

**Table 1:** Crystal data and structure refinement parameters for the BER solid forms obtained

Compound	1BER-1MA	1BER-1MA-1	1BER-2MA-2W	1BER-1LTA-1W	1BER-1DTA-1W	2BER-2CA
Chemical formula	C_20_H_18_NO_4_·C_3_H_3_O_4_	C_20_H_18_NO_4_·C_3_H_3_O_4_	C_20_H_18_NO_4_·C_3_H_4_O_4_·C_3_H_3_O_4_·2(H_2_O)	C_20_H_18_NO_4_·C_4_H_5_O_6_·H_2_O	C_20_H_18_NO_4_·C_4_H_5_O_6_·H_2_O	2C_20_H_18_NO_4_·2C_6_H_7_O_7_
Formula weight	439.41	439.41	579.50	503.45	503.45	1054.92
Crystal system	Monoclinic	Monoclinic	Triclinic	Monoclinic	Monoclinic	Triclinic
Space group	*P*2_1_ *c*	*P*2_1_ *c*	*P* 1	*P*2_1_	*P*2_1_	*P* 1
*a* (Å)	6.844 (1)	6.943 (1)	7.587 (1)	12.111 (1)	12.106 (1)	7.585 (1)
*b* (Å)	19.545 (1)	19.818 (2)	12.937 (1)	7.093 (1)	7.090 (1)	14.686 (1)
*c* (Å)	14.650 (1)	14.669 (2)	13.964 (1)	12.703 (1)	12.699 (1)	22.789 (1)
α (°)	90	90	71.32 (1)	90	90	101.25 (1)
β (°)	98.71 (1)	97.08 (1)	83.35 (1)	99.43 (1)	99.39 (1)	97.33 (1)
γ (°)	90	90	81.36 (1)	90	90	100.89 (1)
*V* (Å^3^)	1937.2(1)	2003.1(3)	1280.4 (2)	1076.6 (1)	1075.5 (1)	2409.2 (2)
*Z*	4	4	2	2	2	2
ρ_calc_ (g cm^−3^)	1.507	1.457	1.503	1.553	1.555	1.451
Radiation	Mo *K*α (λ = 0.71073)	Mo *K*α (λ = 0.71073)	Mo *K*α (λ = 0.71073)	Cu *K*α (λ = 1.54178)	Cu *K*α (λ = 1.54178)	Cu *K*α (λ = 1.54178)
Measured reflections	4584	3526	5902	4170	4305	9389
Independent reflections	4584	2657	5902	4090	4305	9389
Flack parameter	–	–	–	0.05 (6)	0.11 (4)	–
*R*_int_	0.0328	0.0419	0.0339	0.0251	0.0187	0.0463
*R*_1_ [*I* > 2σ(*I*)]	0.1341	0.0992	0.0545	0.0298	0.0257	0.080
*wR*_2_ [*I* > 2σ(*I*)]	0.2788	0.2330	0.1204	0.0831	0.0670	0.226
*R*_1_(all)	0.1417	0.1182	0.0760	0.0302	0.0262	0.1073
*wR*_2_(all)	0.2818	0.2413	0.1399	0.0836	0.0673	0.2353
Goodness of fit	1.049	1.081	1.038	1.076	1.050	1.03
Temperature (K)	100	293	100	100	100	100
Completeness (%)	100	99.0	99.9	95.7	98.8	95.7
CCDC code	2132141	2132142	2132143	2132145	2132144	2132146

**Table 2:** Hydrogen-bond geometry of the BER solid forms obtained

Compound	Hydrogen bond	*D*—H (Å)	H⋯*A* (Å)	*D*⋯*A* (Å)	*D*—H⋯*A* (°)	Symmetry codes
1BER-1MA	O_5_—H_5A_⋯O_7_	0.800 (10)	1.68 (7)	2.434 (6)	156 (19)	
1BER-2MA-2W	O_5_—H_5A_⋯O_13_	1.00 (3)	1.61 (3)	2.5876 (19)	163 (3)	−*x* + 1, −*y* + 1, −*z*
	O_8_—H_8A_⋯O_9_	0.92 (3)	1.63 (3)	2.554 (2))	175 (3)	
	O_10_—H_12C_⋯O_12_	1.20 (3)	1.23 (3)	2.4008 (17)	163 (3)	
	O_13_—H_13C_⋯O_11_	0.86 (3)	1.94 (3)	2.792 (2)	173 (3)	−*x* + 1, −*y* + 1, −*z* − 1
	O_13_—H_13D_⋯O_14_	0.92 (3)	1.84 (3)	2.735 (2)	165 (2)	−*x* + 2, −*y* + 1, −*z* − 1
	O_14_—H_14B_⋯O_6_	0.82 (3)	2.03 (3)	2.833 (2)	166 (3)	−*x* + 2, −*y*, −*z*
	O_14_—H_14C_⋯O_12_	0.95 (4)	1.92 (4)	2.849 (2)	164 (3)	−*x* + 2, −*y*, −*z* − 1
1BER-1LTA-1W	O_7_—H_7A_⋯O_11_	0.87 (4)	1.79 (4)	2.650 (2)	171 (3)	
	O_8_—H_8A_⋯O_10_	0.88 (4)	2.07 (4)	2.643 (2)	122 (3)	
	O_9_—H_9B_⋯O_6_	0.813 (14)	1.654 (15)	2.463 (2)	173 (4)	*x*, *y* + 1, *z*
	O_11_—H_11A_⋯O_5_	0.99 (4)	1.83 (4)	2.816 (2)	173 (3)	*x*, *y* + 1, *z*
	O_11_ —H_11B_⋯O_7_	0.86 (3)	2.21 (3)	2.970 (2)	147 (3)	−*x* + 1, *y* + 1/2, −*z* + 2
1BER-1DTA-1W	O_7_—H_7A_⋯O_11_	0.89 (3)	1.76 (3)	2.650 (2)	175 (3)	−*x*, *y* + 1/2, −*z*
	O_8_—H_8A_⋯O_10_	0.89 (4)	2.06 (4)	2.642 (2)	122 (3)	
	O_9_—H_9B_⋯O_6_	0.821 (14)	1.644 (15)	2.4593 (19)	172 (5)	*x*, *y* − 1, *z*
	O_11_—H_11A_⋯O_5_	0.89 (3)	1.93 (3)	2.816 (2)	171 (3)	*x*, *y* − 1, *z*
	O_11_—H_11B_⋯O_7_	0.84 (3)	2.23 (3)	2.9714 (19)	147 (3)	−*x*, *y* − 3/2, −*z*
2BER-2CA	O_6A_—H_6A_⋯O_9A_	0.872 (19)	1.62 (2)	2.481 (4)	167 (5)	
	O_6B_—H_6B_⋯O_9B_	0.87 (2)	1.67 (2)	2.538 (4)	173 (8)	
	O_7A_—H_7A_⋯O_8A_	0.86 (2)	2.18 (7)	2.613 (5)	111 (6)	
	O_7B_—H_7B_⋯O_8B_	0.859 (19)	2.10 (4)	2.604 (3)	117 (4)	
	O_10A_—H_10A_⋯O_8A_	0.85	1.92	2.758 (5)	166.6	*x* − 1, *y*, *z*
	O_10B_—H_10B_⋯O_9B_	0.853 (10)	1.693 (11)	2.545 (3)	176 (5)	*x* − 1, *y*, *z*

**Table 3:** Water molecule loss numbers and dehydration temperature of six BER salts

	First peak (K)	Weight loss (%)	Second peak (K)	Weight loss (%)	Third peak (K)	Weight loss (%)	Fourth peak (K)	Weight loss (%)	Observed stoichiometry	Calculated stoichiometry
1BER-1MA	470.7	20.55	–	–	–	–	–	–	–	–
1BER-2MA-2W	352.0	6.20	391.5	17.80	418.8	–	467.7	16.6	1:2	1:2
1BER-1L-TA-1W	375.7	3.55	498.2	26.88	–	–	–	–	1:1	1:1
1BER-1D-TA-1W	379.9	3.65	497.8	27.78	–	–	–	–	1:1	1:1
1BER-1DL-TA	507.8	29.48	–	–	–	–	–	–	–	–
2BER-2CA	481.3	26.5	–	–	–	–	–	–	–	–

**Table 4:** Stability of the synthesized products kept at high humidity and high temperature

	HT-313K-10d	HT-333K-10d	HH-90%-10d
1BER-1MA	–	1BER-1MA	1BER-1MA
1BER-2MA-2W	1BER-2MA-2W	1BER-2MA-2W-AH	1BER-2MA-2W
1BER-1LTA-1W	–	1BER-1LTA-1W	1BER-1LTA-1W
1BER-1DTA-1W	–	1BER-1DTA-1W	1BER-1DTA-1W
1BER-1DLTA	–	1BER-1DLTA	1BER-1DLTA
1BER-1CA	–	1BER-1CA	1BER-1CA

**Table 5:** Solid forms of undissolved samples in the solubility experiment

Pure water			Dilute hydro­chloric solution		
0 h	3 h	48 h	0 h	3 h	48 h
1BER-1HCl-M	1BER-1HCl-4W	1BER-1HCl-4W	1BER-1HCl-M	1BER-1HCl-4W	1BER-1HCl-4W
1BER-1MA	1BER-1MA	1BER-1MA	1BER-1MA	1BER-1HCl-4W	1BER-1HCl-4W
1BER-2MA-2W	1BER-2MA-2W	1BER-2MA-2W	1BER-2MA-2W	1BER-1HCl-4W	1BER-1HCl-4W
1BER-1D-TA-1W	1BER-1D-TA-1W	1BER-1D-TA-1W	1BER-1D-TA-1W	1BER-1D-TA-1W + 1BER-1HCl-4W	1BER-1HCl-4W
1BER-1L-TA-1W	1BER-1L-TA-1W	1BER-1L-TA-1W	1BER-1L-TA-1W	1BER-1L-TA-1W + 1BER-1HCl-4W	1BER-1HCl-4W
1BER-1DL-TA	1BER-1DL-TA	1BER-1DL-TA	1BER-1DL-TA	1BER-1DL-TA	1BER-1HCl-4W
1BER-1CA	1BER-1CA	1BER-1CA	1BER-1CA	1BER-1HCl-4W	1BER-1HCl-4W
