# Supplementary material for: Novel multi-component crystals of berberine with improved pharmaceutical properties
Source: IUCrJ. 2023 Jan 1;10(Pt 1):66–76. doi: 10.1107/S2052252522010983 (PMC9812220; doi:10.1107/S2052252522010983)
Supplement: Supplementary file 14 [file m-10-00066-sup14.pdf]

IUCrJ

Volume 10 (2023)

Supporting information for article:

Novel multi-component crystals of berberine with improved pharmaceutical properties

Guoshun Zhang, Xirui Yang, Xiaoqing Shang, Wei Han, Fengfeng Wang, Shurong Ban and Shuqiu Zhang

Table 1. Reactive crystallization experiments of 8H-HBER and MA in different mixed solvents

8H-HBER/MA(molar ratio)	V(ml)/V(ml)	2/0	1.8/0.2	1.8/0.4	1.4/0.6	1.0/1.0	0.6/1.4
1:1	Ethanol -water	1	1	1	1	1	1
1:1	Acetone-water	*	2	2	2	2	2
1:2	Ethanol -water	1	1	2	2	2	2
1:2	Acetone-water	*	2	2	2	2	2

Note: 1 and 2 represent 1BER-1MA and 1BER-2MA-2W, respectively. *: The experiment was not carried out due to the low solubility of the reactants in the solvent system.

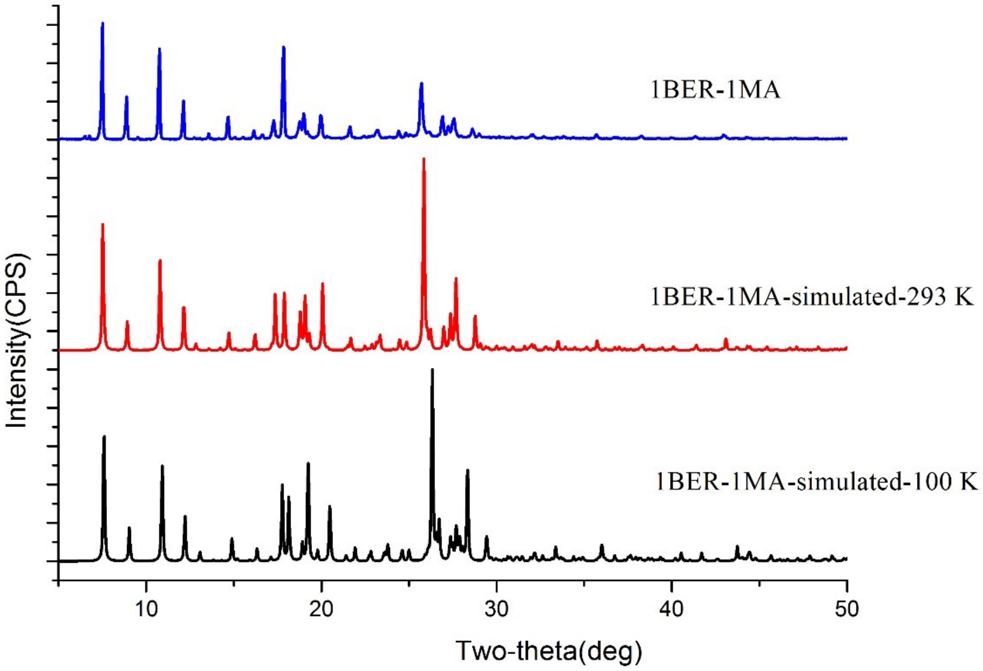

Figure 1 The experiment and calculated PXRD patterns of 1BER-1MA

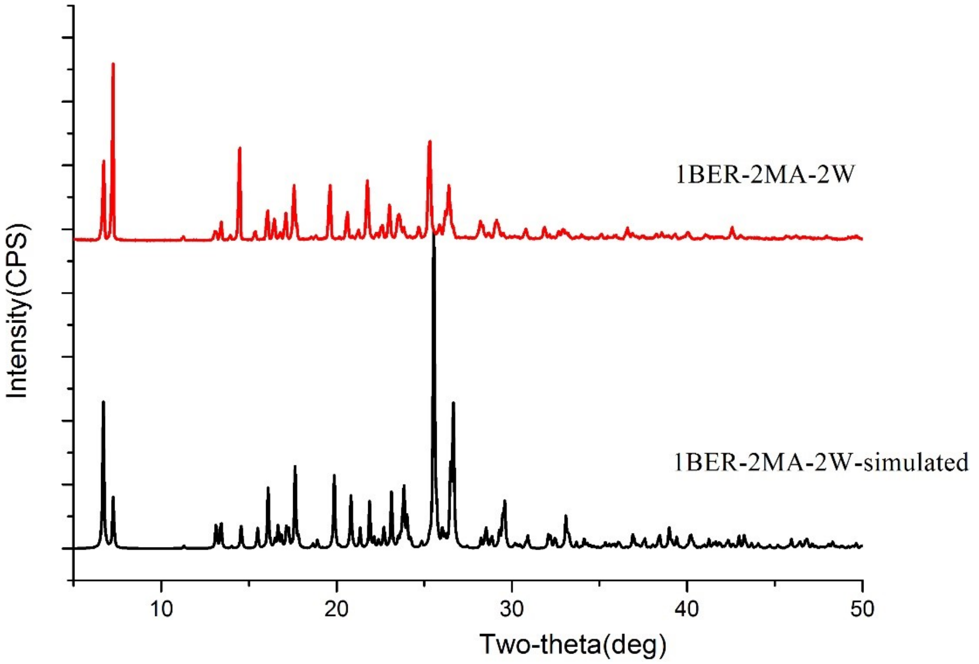

Figure 2 The experiment and calculated PXRD patterns of 1BER-2MA-2W

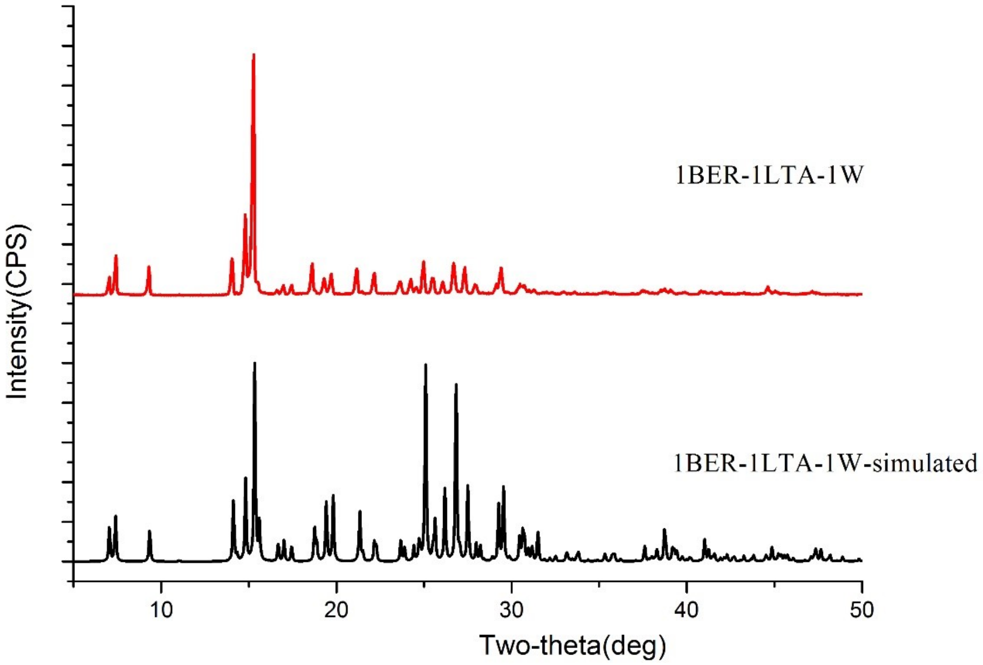

Figure 3 The experiment and calculated PXRD patterns of 1BER-1LTA-1W

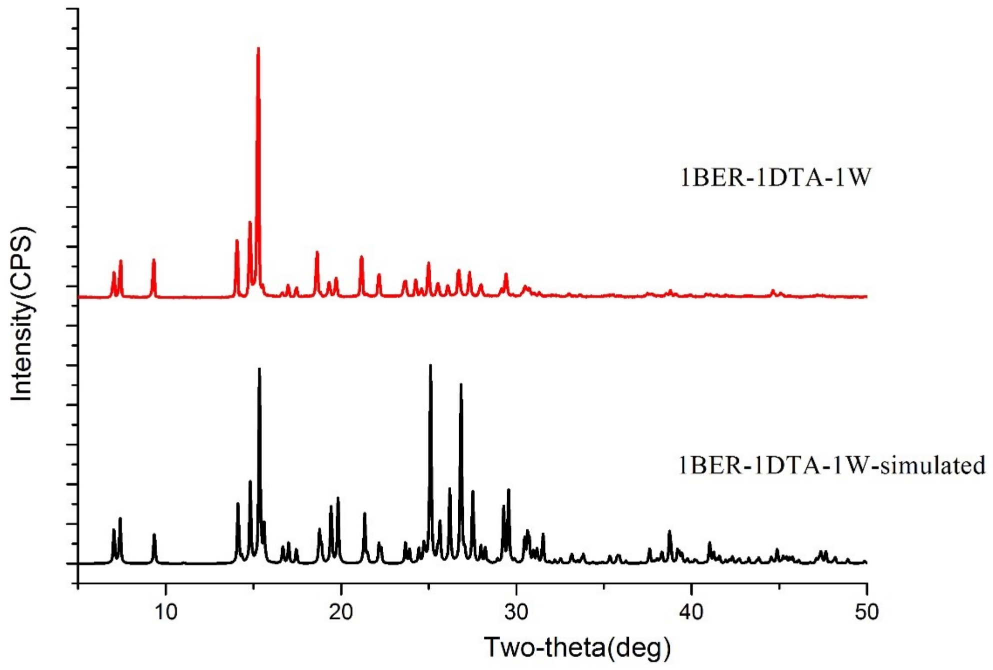

Figure 4 The experiment and calculated PXRD patterns of 1BER-1DTA-1W

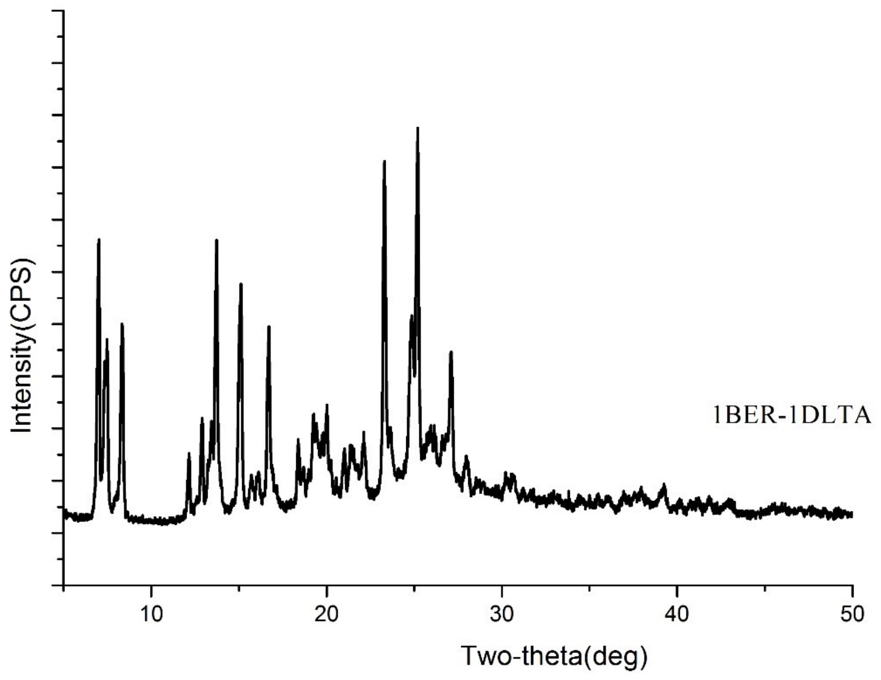

Figure 5 The experiment PXRD patterns of 1BER-1DLTA

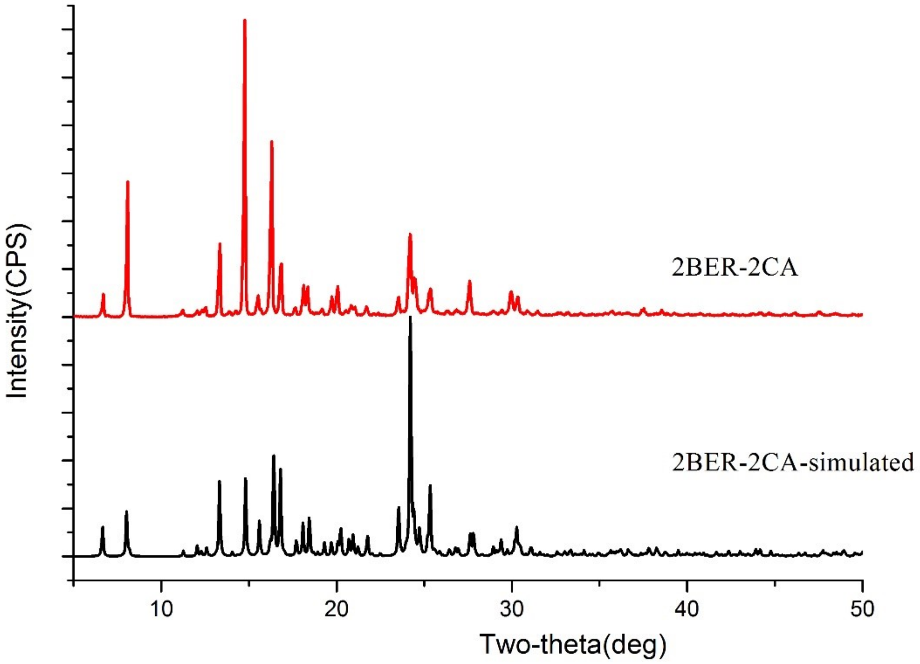

Figure 6 The experiment and calculated PXRD patterns of 2BER-2CA

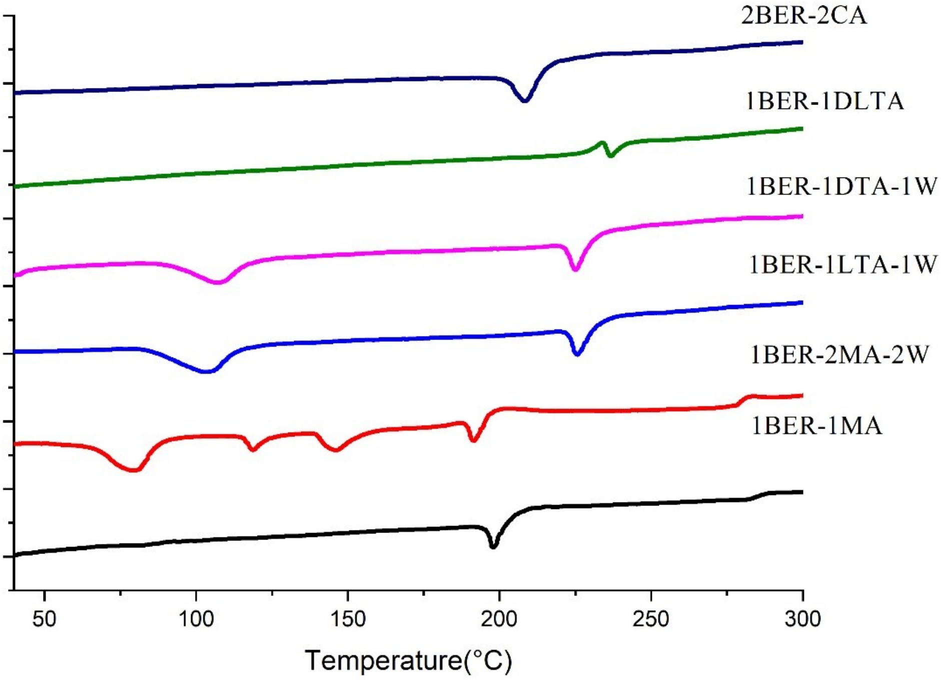

Figure 7 DSC profiles of six kinds of the BER related solid forms

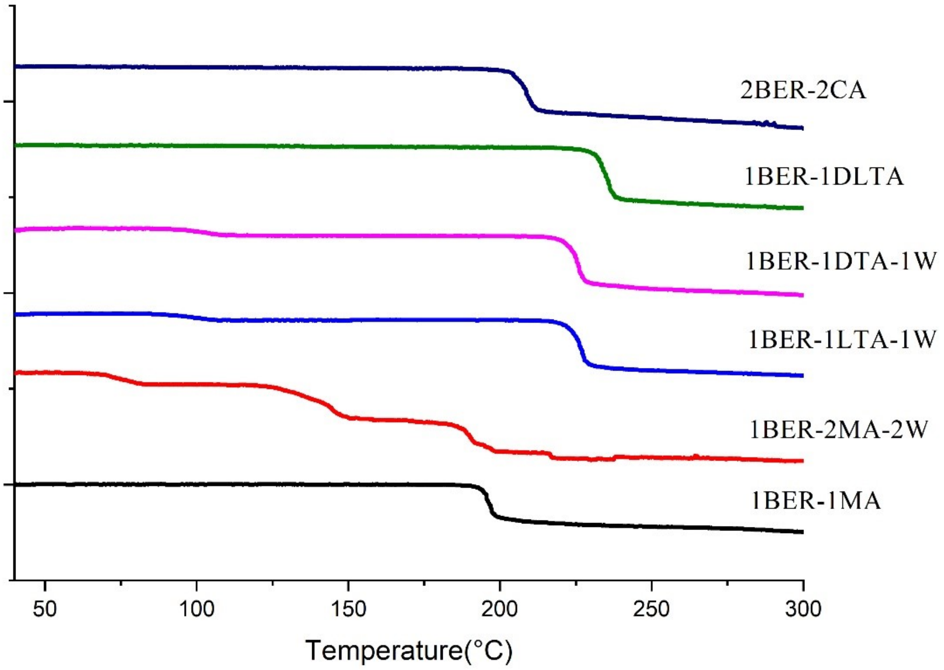

Figure 8 TGA profiles of six kinds of the BER related solid forms

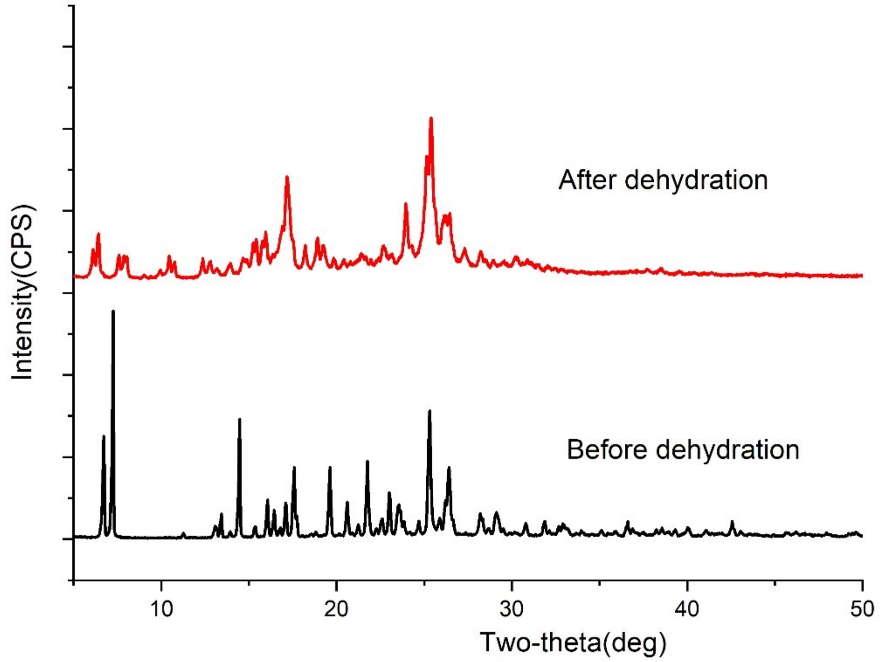

Figure 9 The experiment PXRD patterns of 1BER-2MA-2W before and after dehydration

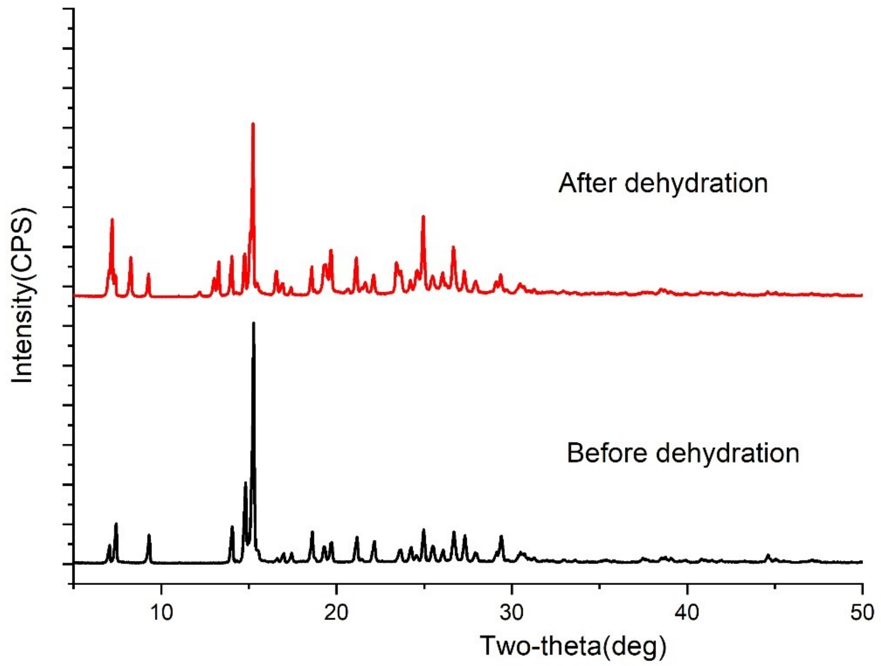

Figure 10 The experiment PXRD patterns of 1BER-1LTA-1W before and after dehydration

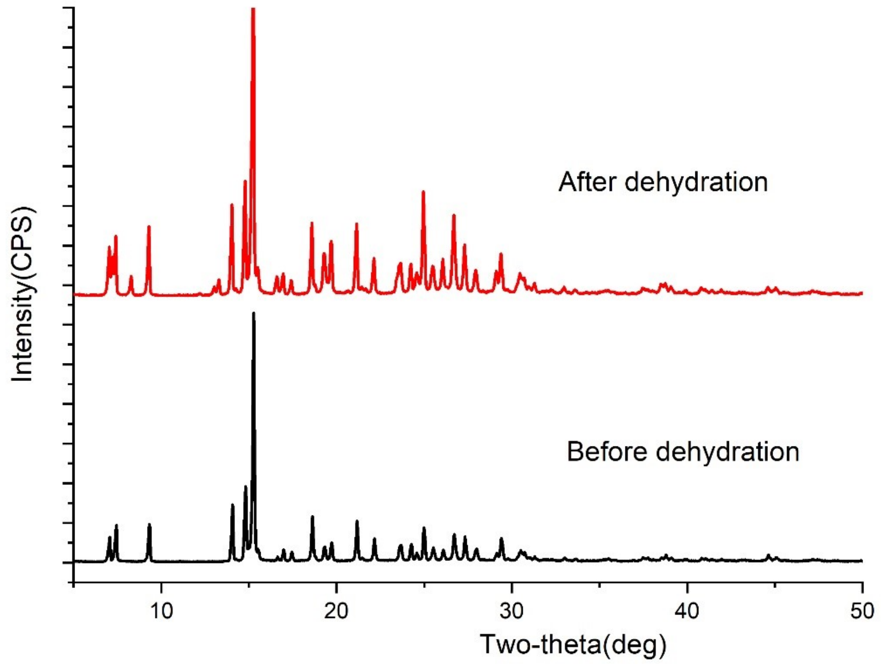

Figure 11 The experiment PXRD patterns of 1BER-1DTA-1W before and after dehydration

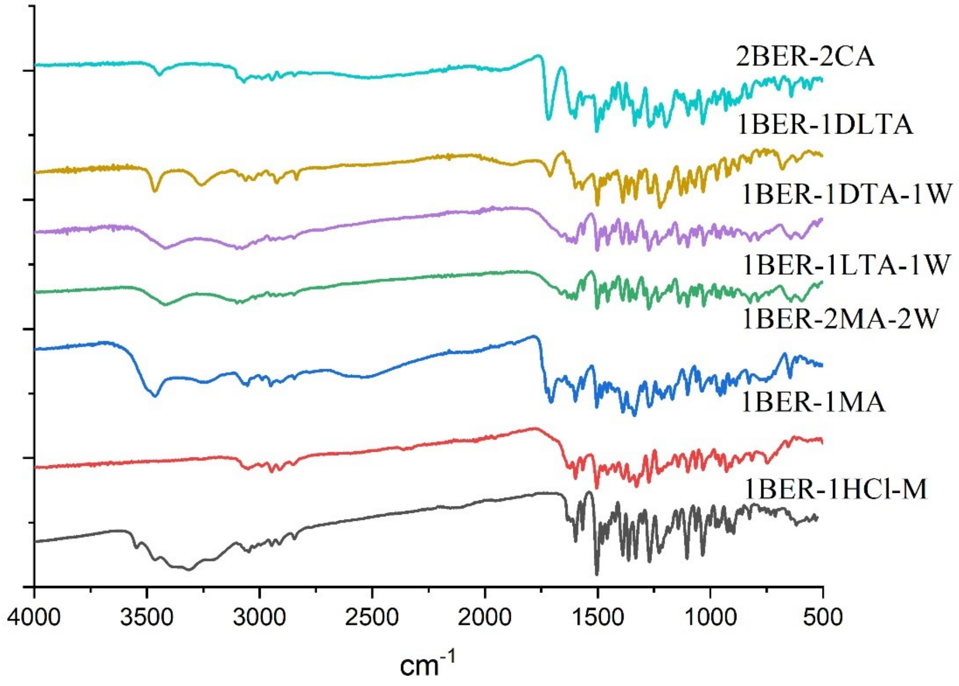

Figure 12 FT-IR profiles of seven kinds of the BER related solid forms

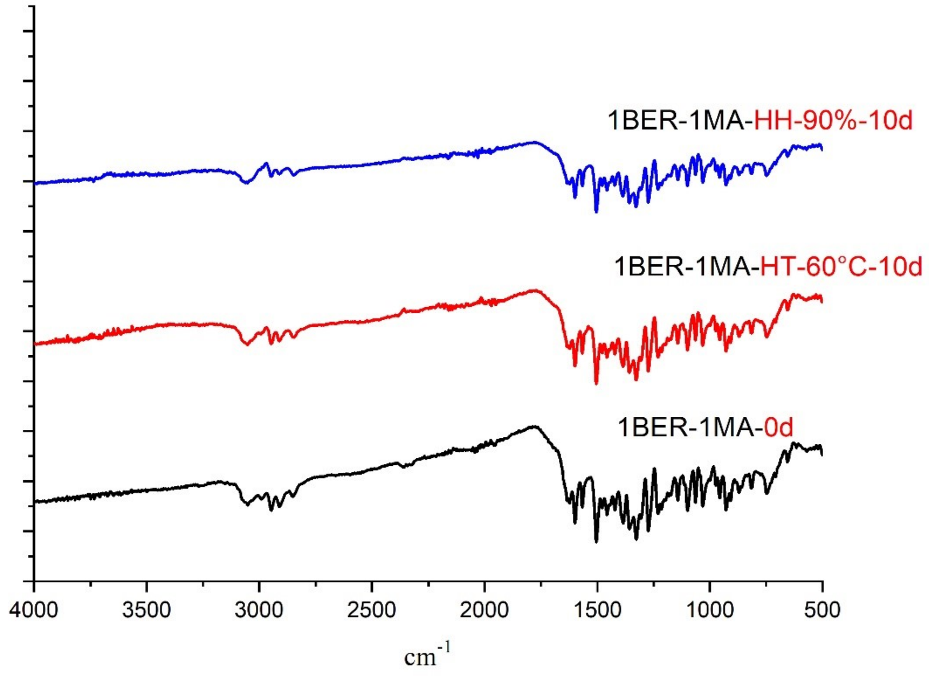

Figure 13 FT-IR profiles of stability of 1BER-1MA

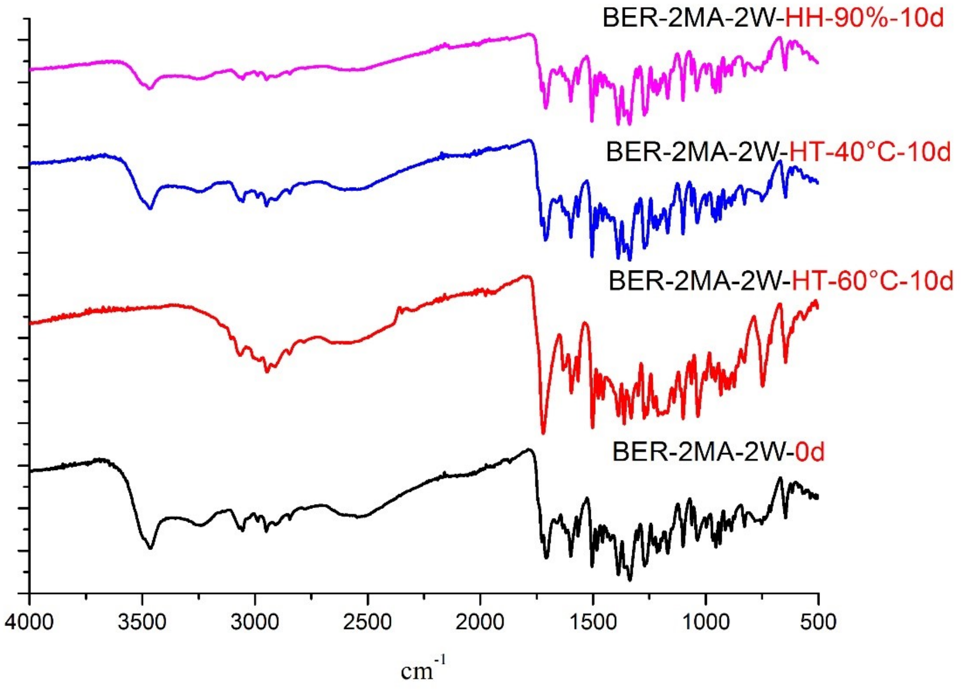

Figure 14 FT-IR profiles of stability of 1BER-2MA-2W

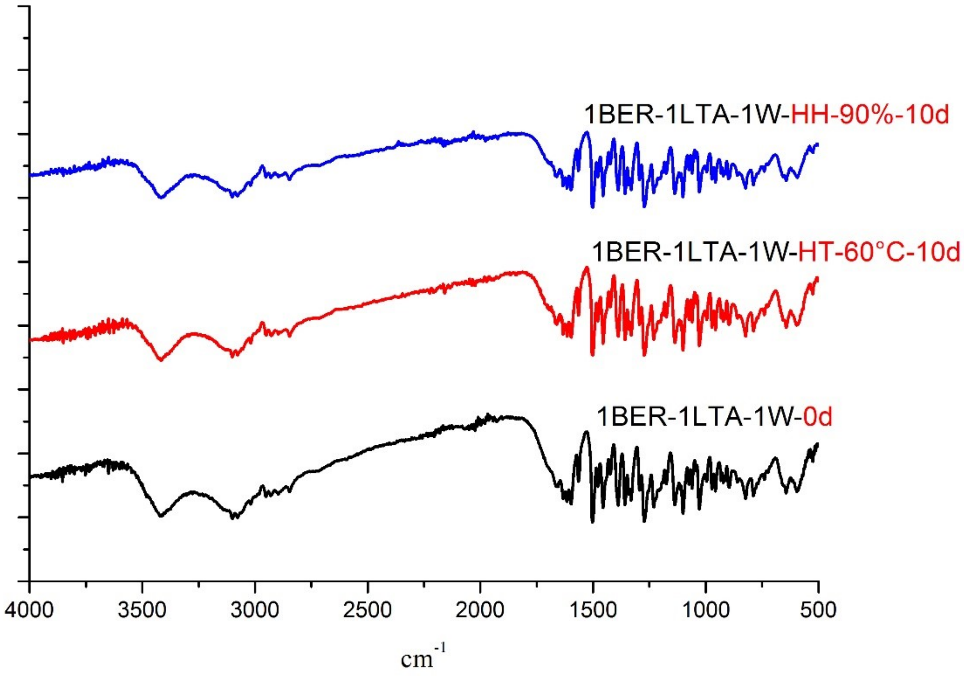

Figure 15 FT-IR profiles of stability of 1BER-1LTA-1W

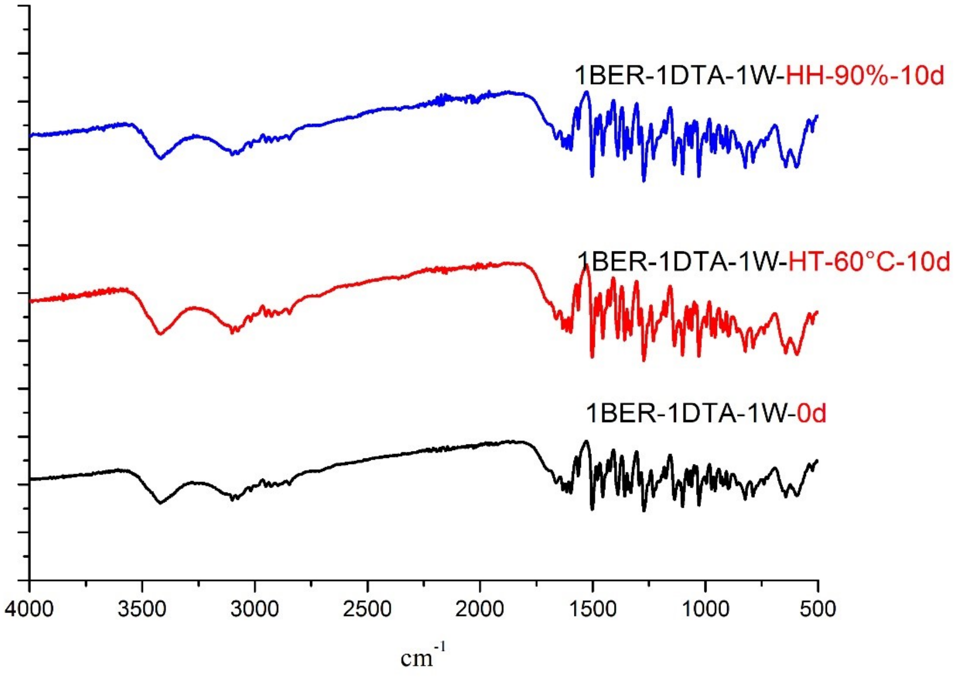

Figure 16 FT-IR profiles of stability of 1BER-1DTA-1W

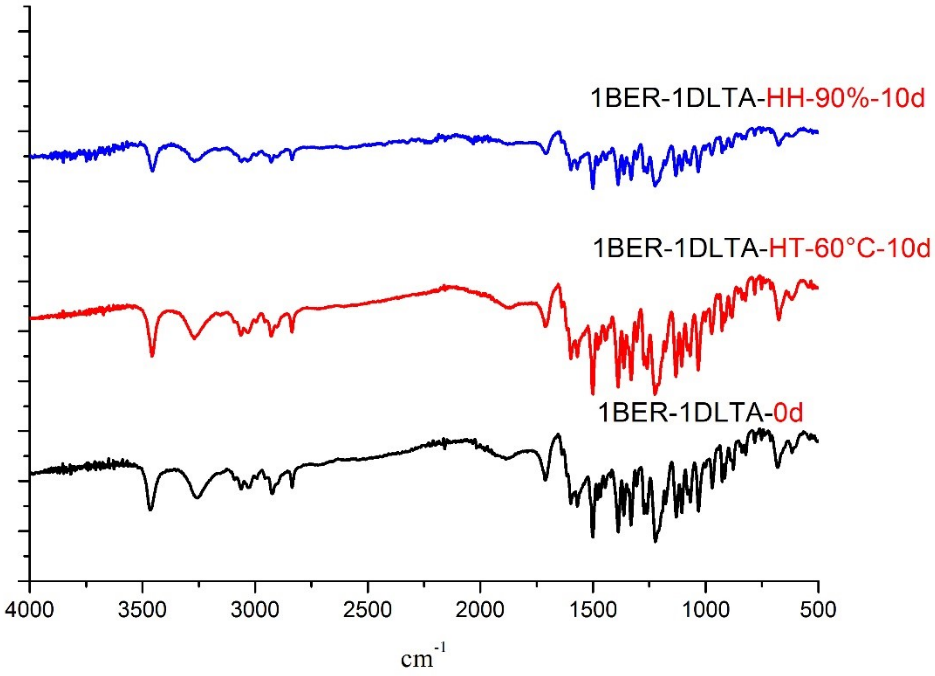

Figure 17 FT-IR profiles of stability of 1BER-1DLTA

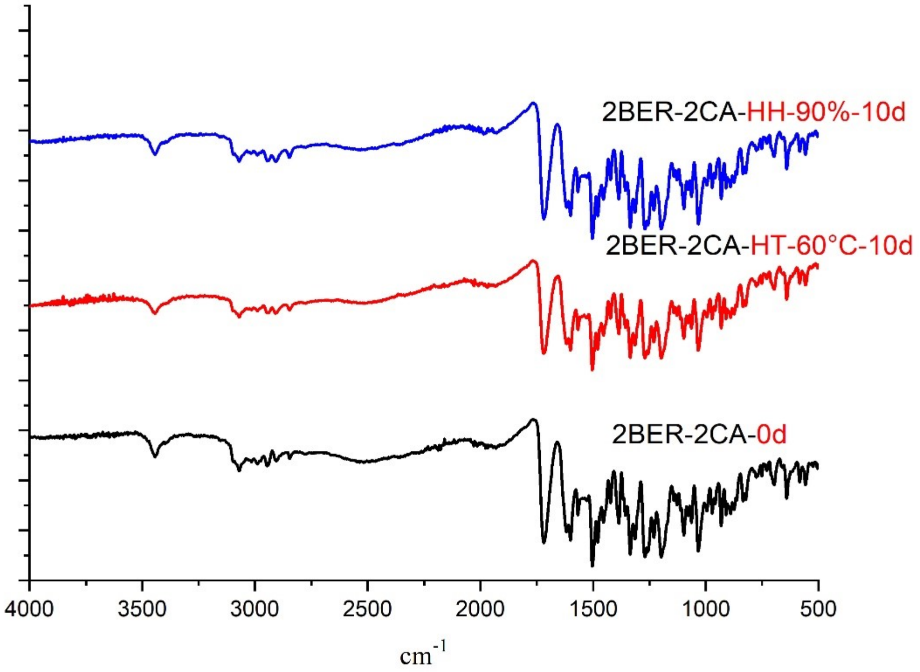

Figure 18 FT-IR profiles of stability of 2BER-2CA

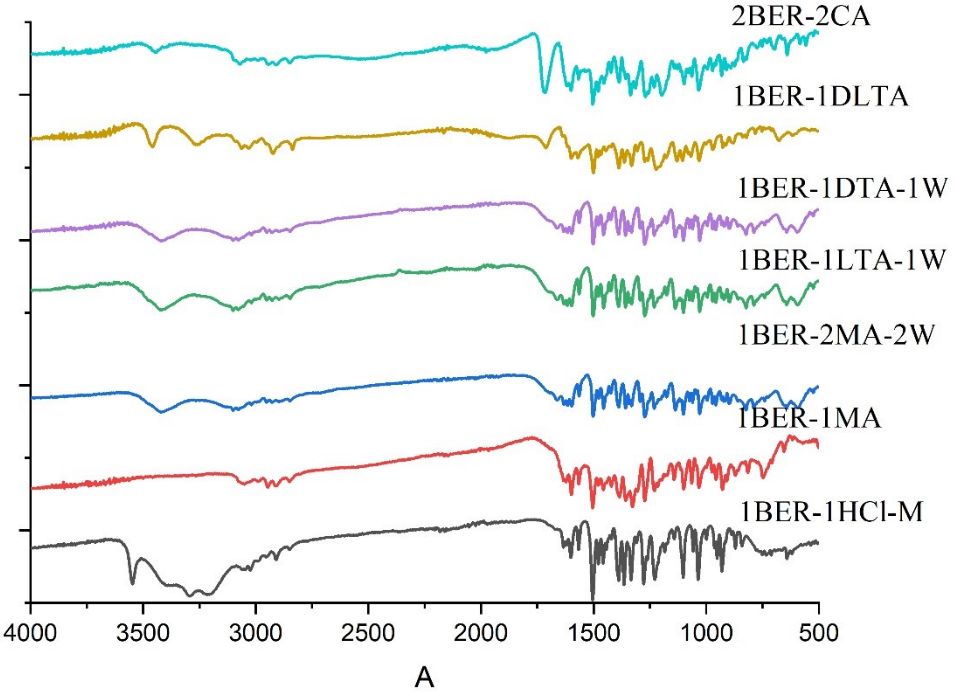

Figure 19. FT-IR profiles of the undissolved solid phase forms in pure water at 3 hours

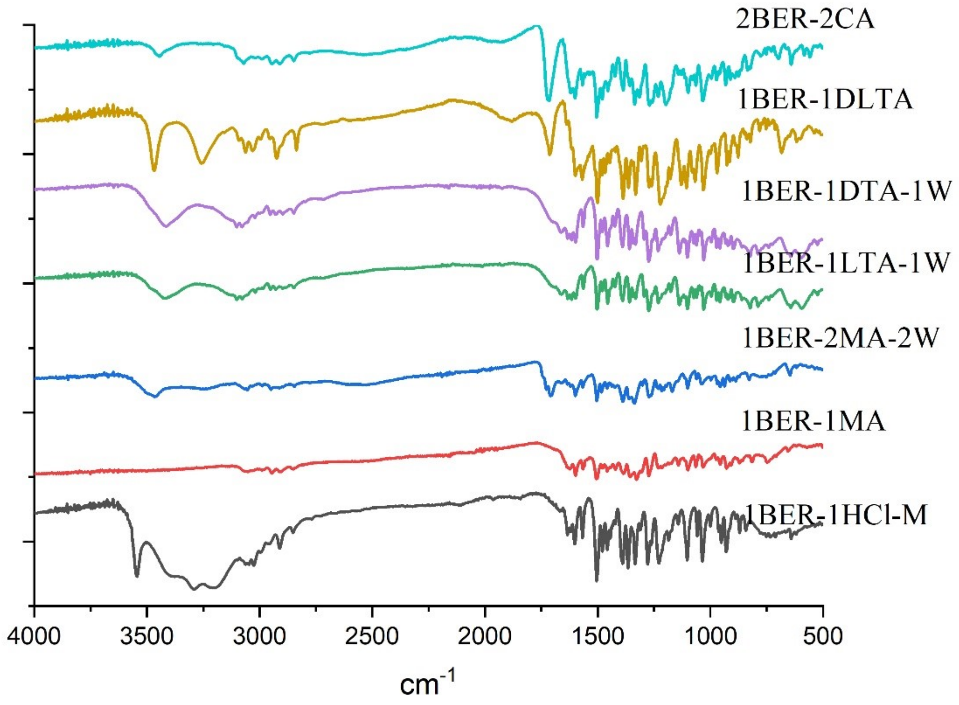

Figure 20. FT-IR profiles of the undissolved solid phase forms in pure water at 48 hours

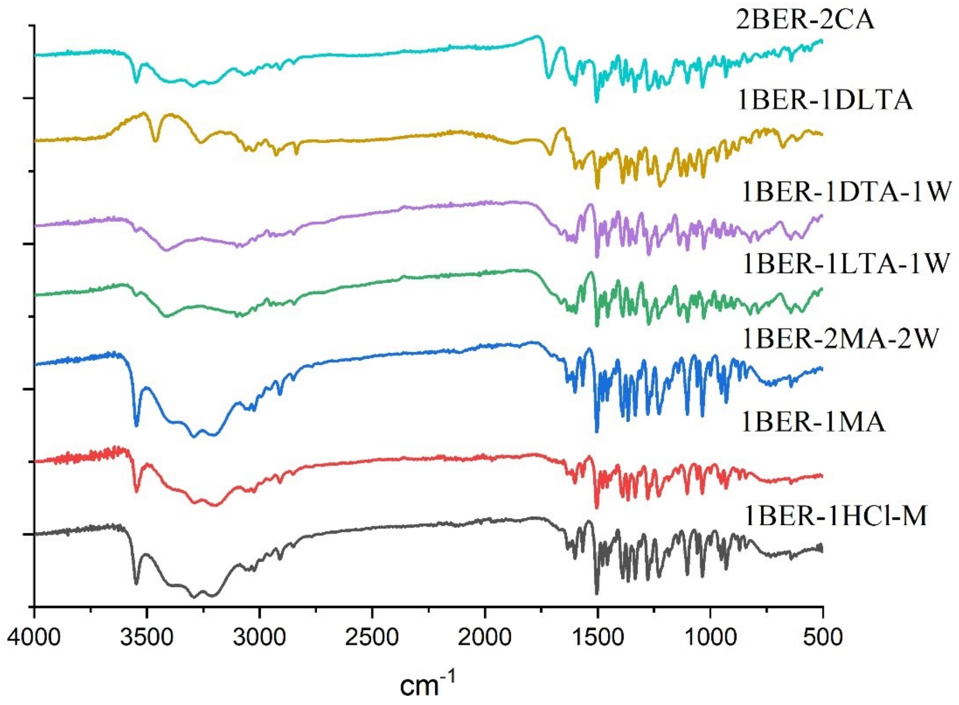

Figure 21. FT-IR profiles of the undissolved solid phase forms in dilute hydrochloric acid solution at 3 hours

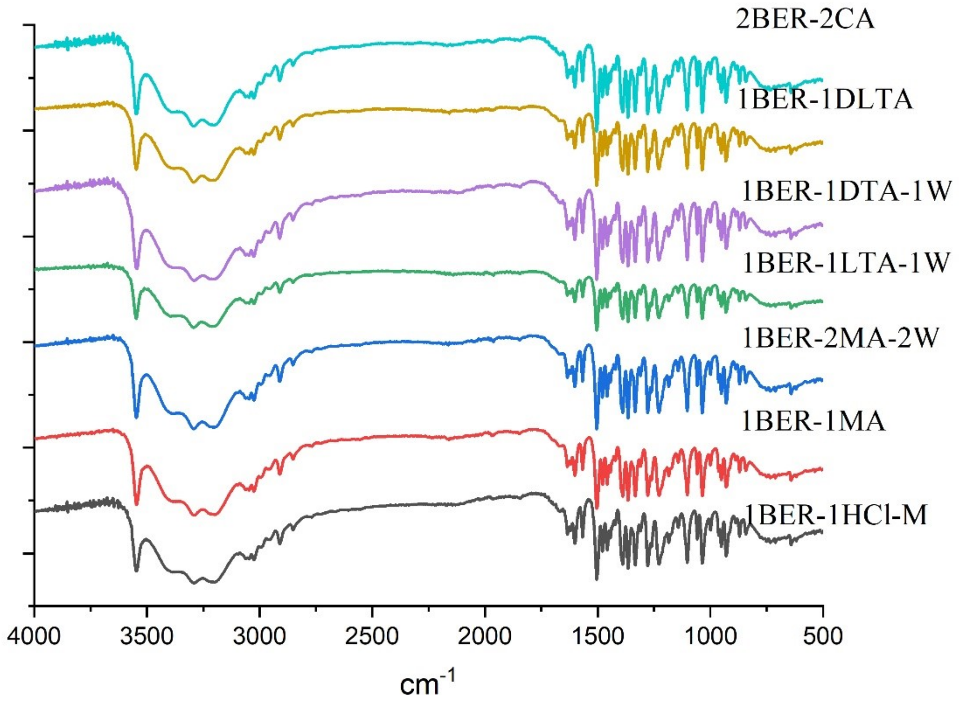

Figure 22. FT-IR profiles of the undissolved solid phase forms in dilute hydrochloric acid solution at 48 hours
